# Resting-State Functional Connectivity Patterns Predict Chinese Word Reading Competency

**DOI:** 10.1371/journal.pone.0044848

**Published:** 2012-09-24

**Authors:** Xiaosha Wang, Zaizhu Han, Yong He, Li Liu, Yanchao Bi

**Affiliations:** State Key Laboratory of Cognitive Neuroscience and Learning, Beijing Normal University, Beijing, China; Indiana University, United States of America

## Abstract

Resting-state functional connectivity (RSFC) offers a novel approach to reveal the temporal synchronization of functionally related brain regions. Recent studies have identified several RSFCs whose strength was associated with reading competence in alphabetic languages. In the present study, we examined the role of intrinsic functional relations for reading a non-alphabetic language – Chinese – by correlating RSFC maps of nine Chinese reading-related seed regions and reaction time in the single-character reading task. We found that Chinese reading efficiency was positively correlated with the connection between left inferior occipital gyrus and left superior parietal lobule, between right posterior fusiform gyrus and right superior parietal lobule, and between left inferior temporal gyrus and left inferior parietal lobule. These results could not be attributed to inter-individual differences arising from the peripheral processes of the reading task such as visual input detection and articulation. The observed RSFC-reading correlation relationships are discussed in the framework of Chinese character reading, including visuospatial analyses and semantic/phonological processes.

## Introduction

Oral word reading is a learned complex cognitive skill that entails orthographic access and subsequent activation of word phonology and meaning. On the one hand, much universality has been shown in cognitive and neural mechanisms of reading across languages, such as the existence of dual routes for reading aloud: one is via lexical semantics, i.e., the visual input activates its representation in the orthographic lexicon which activates the lexical semantic representation, and then the phonological representation that finally leads to the phonetic and articulation programming. In the other route, the phonological representation is activated by the orthographic input through direct mapping. These two routes may function in a highly interactive manner (e.g., [1]–[3]). On the other hand, differences have also been revealed for both representational and dynamic aspects of reading across scripts, especially between alphabetic and non-alphabetic languages [4], [5].

One typical example is Mandarin Chinese, a logographic system that differs from alphabetic scripts in many important aspects, including the lack of grapheme-phoneme correspondence and greater visual complexity inherent in logographic characters. Meta-analysis studies comparing Chinese and alphabetic languages have identified several cortical regions that are activated both by Chinese and English reading tasks, including left occipitotemporal cortex and left inferior frontal gyrus [4], [5] as well as the anterior and lateral regions of left superior temporal gyrus [4]. Critically, however, some regions have been reported to be consistently activated in reading Chinese but not or less in reading English: right occipitotemporal regions [4]–[7] and left middle frontal gyrus or the adjacent dorsal inferior frontal lobe regions ([4], [5]; but see [8]). Such differences have been attributed to specific cognitive processes associated with complex visuospatial analyses of Chinese characters and with addressed phonology in the Chinese script, respectively.

An approach that examines the functional relevance of intrinsic brain activity patterns, namely the resting-state functional connectivity (RSFC)-behavior correlation analysis, has recently been applied to investigate neural mechanisms of human behaviors. Studies have shown that spontaneous fluctuation in the blood-oxygenation level-dependent (BOLD) signals in functionally related brain regions are temporally correlated, indicating the synchronization of intrinsic neuronal activity among these brain regions [9], [10]. Importantly, correlating the RSFC strength with behavioral performance can provide more direct evidence about the role of RSFC in certain cognitive skills. So far, the RSFC-behavior correlation analysis has been used to investigate neural bases of a wide range of cognitive functions, including working memory [11], perceptual learning [12], executive control [13], personality traits [14], [15] and intelligence [16].

Specifically, two recent studies have applied this approach to explore neural mechanisms of English reading. The first study [17] looked at two specific regions that have been consistently implicated in reading: left Brodmann area 39 (BA 39, including the angular gyrus and the superior posterior aspect of the medial temporal gyrus) and Broca's area. They found that reading performance was significantly correlated with the functional connectivity strength between these two regions both during task-related and resting-state fMRI, although to a lesser extent for the latter. Another study by Koyama et al. (2011) compared RSFC-reading correlation patterns between children and adults [18]. They used 11 reading-related seeds derived from task-based fMRI studies to construct whole-brain RSFC maps, among which the RSFC between left precentral gyrus and other motor regions, and between Broca's and Wernicke's areas were found to be positively correlated with reading competence in both children and adults. Moreover, in comparison to children, adults with better reading performance showed stronger positive correlations between left fusiform gyrus (FFG) and phonology-related regions and stronger negative correlations between FFG and regions in the default mode network. These results highlighted the significance of motor regions, the Broca's area-Wernicke's area pathway and FFG-related circuitry for the efficient and automatized reading in adult readers of English.

In light of these recent findings in English reading, the current study explored the role of intrinsic inter-regional connections for Chinese reading by examining the relationship between RSFCs and reading competence of Chinese speakers. Specifically, we examined the RSFCs of nine cortical seed regions that were consistently involved in Chinese reading tasks [4]. For each seed region, we generated its grey-matter RSFC map and then computed correlations between the RSFC map and participants' performances in a single-character reading task. To explicitly examine the language specificity issue, we further carried out a post hoc analysis on RSFCs observed with English speakers reported by Koyama et al. (2011) so as to assess the extent to which these interregional connections could predict reading performance of our Chinese participants.

## Materials and Methods

### Participants

All experiments and procedures were approved by the Institutional Review Board of the State Key Laboratory of Cognitive Neuroscience and Learning, Beijing Normal University. Written consent was obtained from each participant. Thirty-two right-handed young healthy adults recruited from Beijing Normal University (13 male, mean age = 22.4±1.3 years, age range = 20–26 years) participated in the current study. All participants were native Chinese speakers and had reported no history of any psychiatric or neurological diseases. Fourteen of the 32 subjects in the current study participated in the IQ test (mean full IQ 125.79±7.14, range = 115–133) [19].

### Behavioral tasks

The main task to assess participants' reading competence was single-character reading. The stimuli set was taken from a previous reading study [2], which contained 150 single-syllable characters covering a wide range in both frequency (range = 7–975 per 1.8 million) and visual complexity (number of strokes range = 5–15). All characters were so-called composite characters comprising of a phonetic radical and a semantic radical and these radicals were either transparent or opaque. Transparent radicals refer to the cases where the phonetic or semantic radical provides cues for the pronunciation or meaning of the whole character, whereas opaque radicals meant that no such relationship is implied. In each trial, a fixation cross was presented at the center of the screen for 500 ms and then replaced by a target character at font SONG, size 36. The character disappeared either upon a vocal response by participants or after a two-second deadline. The next trial started one second later. All characters were presented in a pseudo-random order such that phonologically similar characters did not occur on consecutive trials. Participants were instructed to read the characters aloud as quickly and accurately as possible. Response accuracy was coded by the experimenter; erroneous responses, no responses, stuttering were coded as errors. The whole task was completed in about 10 minutes.

In addition, one control task was administered, namely the cued articulation task. This task was done to assess individual differences arising from basic input and output characteristics of the reading paradigm and thus to rule out the possibility that the potential interregional RSFCs underlying reading are due to some basic cognitive skills such as visual input detection and articulation. In this task, participants were asked to pronounce a sound “ah” as soon as they saw the fixation cross. The detailed trial structure was identical to that of the single-character reading task except that a random trial interval, i.e. 500, 1000 or 2000 ms, was adopted to prevent participants from resorting to the anticipation strategy. There were 20 trials in total.

The DMDX program was used to present stimuli and record reaction time of vocal production via a microphone [20]. These behavioral data were acquired about one year after the imaging scanning.

### fMRI data acquisition

MRI data were collected on a SIEMENS TRIO 3-Tesla scanner at the Imaging Center for Brain Research, Beijing Normal University. For resting-state functional imaging, scans lasted about eight minutes and were composed of 240 continuous echo-planar imaging (EPI) whole-brain functional volumes (time repetition (TR) = 2000 ms; time echo (TE) = 30 ms; flip angle = 90°; 33 axial slices; matrix = 64×64; field of view (FOV) = 200×200 mm^2^; acquisition voxel size = 3.1×3.1×3.5 mm^3^). During the scan, participants were instructed to relax with their eyes closed and try not to think about anything systematically or fall asleep. None of them fell asleep according to a simple questionnaire after the scan. For spatial normalization and localization, a T1-weighted anatomical image was obtained using a magnetization-prepared rapid gradient echo (MPRAGE) sequence (TR = 2530 ms; TE = 3.39 ms; inversion time (TI) = 1100 ms; flip angle = 7°; 128 sagittal slices; FOV = 256×256 mm^2^; voxel size = 1.3×1.0×1.3 mm^3^).

### Seed regions of interest (ROI)

**Figure 1:**
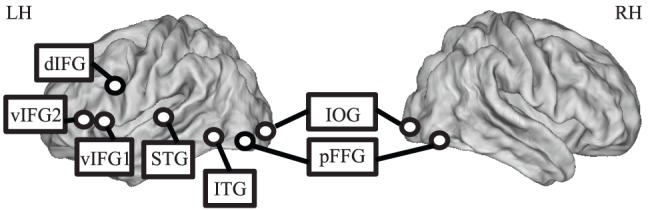
Seed regions-of-interest (ROIs). Nine seed regions were selected from a meta-analysis of Chinese single word reading [4]. LH = left hemisphere; RH = right hemisphere; IOG = inferior occipital gyrus; pFFG = posterior fusiform gyrus; ITG = inferior temporal gyrus; STG = superior temporal gyrus; dIFG = dorsal inferior frontal gyrus; vIFG1 = ventral inferior frontal gyrus seed 1; vIFG2 = ventral inferior frontal gyrus seed 2.

ROIs for the resting-state functional connectivity (RSFC) analysis were selected from a meta-analysis study of single word reading in several languages including Chinese [4]. We focused on this study instead of other Chinese reading meta-analyses because 1) the tasks included in Bolger et al. (2005) entailed a complete reading process, which corresponds to the single-character reading task in our study; 2) the English reading-related ROIs in adult readers in [18] were also taken from Bolger et al. (2005), making the English-Chinese comparison more direct. Bolger et al. (2005) included nine Chinese reading studies that focused on single word recognition, such as word reading, semantic or phonological judgment, and reported nine consistently activated cortical foci across these studies. For each seed, we converted the reported Talairach coordinate into the the Montreal Neurological Institute (MNI) coordinate [21] and created a spherical ROI centering on the MNI coordinate with a radius of 6 mm. Although two seeds in left ventral inferior frontal gyrus were spatially adjacent to each other, we included them both since the Euclidean distance between their MNI coordinates were verified to be larger than 12 mm. See Figure 1 and Table 1 for the exact locations of these ROIs.

**Table 1:** MNI coordinates of nine Chinese-reading related seed regions-of-interest (ROIs).

Seed regions of interest	BA	MNI coordinates		
		x	y	z
*Left hemisphere*				
L.IOG	18	−24	−98	−6
L.pFFG	19	−39	−80	−12
L.ITG	37	−52	−56	−9
L.STG	40	−67	−21	1
L.vIFG1	9	−44	24	2
L.vIFG2	45	−48	36	1
L.dIFG	9	−50	14	29
*Right hemisphere*				
R.IOG	18	33	−94	−6
R.pFFG	19	37	−71	−14

Note: Nine seed regions were selected from a meta-analysis of Chinese single word reading [4]. L = left; R = right; IOG = inferior occipital gyrus; pFFG = posterior fusiform gyrus; ITG = inferior temporal gyrus; STG = superior temporal gyrus; dIFG = dorsal inferior frontal gyrus; vIFG1 = ventral inferior frontal gyrus seed 1; vIFG2 = ventral inferior frontal gyrus seed 2; BA = Brodmann area.

Detailed discussion on the roles of these ROIs in Chinese reading can be found in Bolger et al. (2005)'s study, which are summarized briefly as follows. ROI 1–4: Bilateral occipitotemporal regions, including inferior occipital gyri (L.IOG and R.IOG) and posterior fusiform gyri (L.pFFG and R.pFFG). While left occipitotemporal regions (L.IOG and L.pFFG) are consistently engaged in visual word form processing across different writing systems, their right counterparts are only found to be activated in Chinese, which have been contended to be due to greater visual complexity of Chinese characters. ROI 5: Left inferior temporal gyrus (L.ITG), the so-called visual word form area (VWFA), specialized in processing abstract orthographic representations regardless of variation in visual features and orthographic-phonologic-semantic mapping functions across writing systems ([22], but see [23]). ROI 6: The anterolateral portion of left superior temporal gyrus (L.STG), convergent across writing systems and involved in phonological identification of word form (but see [5]). ROI 7–8: The ventral aspect of left inferior frontal gyrus (L.vIFG1 and L.vIFG2), which are critical to semantic processing. ROI 9: The dorsal aspect of left inferior frontal gyrus (L.dIFG) was assumed in Bolger et al. (2005) to be involved in phonological decision making. It was close to the well-known left middle frontal gyrus that has been described by Tan and colleagues [5] as being specific to Chinese-reading, although the exact relationships are not unequivocal [4].

### Data preprocessing for RSFC analyses

Resting-state MRI data preprocessing was carried out using SPM5 (see http://www.fil.ion.ucl.ac.uk/spm) and Data Processing Assistant for Resting-State fMRI [24] in the following steps: 1) discarding the first 10 volumes for signal equilibrium; 2) slice timing correction; 3) head motion correction; no participant exhibited head motion of 2 mm maximum translation or 2° rotation throughout the course of scans. 4) spatial normalization to the MNI space using T1 image unified segmentation; the resampling voxel size was 3×3×3 mm^3^; 5) spatial smoothing with 6 mm FWHM Gaussian kernel; 6) removal of linear trends; 7) band-pass temporal filtering (0.01–0.1 Hz); 8) removal of several nuisance covariates by regression to control for the effects of physiological processes and head motion (six head motion parameters, the white matter signal, the cerebrospinal fluid signal and the global signal). We also considered results without the global signal removal and the overall result patterns were in the same directions with those with the global signal removed: for the six RSFCs that showed significant correlation with reading competence presented below in the Results section (Table 2), the peak *r* values without the global signal removal were between 0.34 and 0.49 for positive correlations and −0.34–−0.52 for negative correlations (*p*s<0.056).

**Table 2:** MNI coordinates and correlation coefficients of the RSFC-reading correlation relationships.

ROIs	Connected regions	BA	Volume (mm^3^)	Peak (MNI)			*r*-value	
				x	y	z	Direct correlation	Controlled for cued articulation and mean FD
L.IOG	L.SPL	5	1080	−15	−51	60	0.59	0.56
R.pFFG	R.SPL	5/7	972	21	−45	72	0.54	0.53
L.ITG	L.IPL	40/48	783	−60	−51	33	0.56	0.59
L.STG	R.OFC	11	945	27	39	−18	−0.68	−0.72
L.vIFG1	R.THA	–	2079	6	−12	9	−0.60	−0.56
L.vIFG2	B.PCUN	7/–	1458	−9	−54	42	−0.59	−0.54

Note: The *r*-values refer to Pearson's correlation coefficients between RSFC connecting the sphere centering on the peak coordinate of the cluster and the corresponding ROI and reading scores with or without cued articulation scores and mean FD regressed out. L = left; R = right; B = bilateral; IOG = inferior occipital gyrus; SPL = superior parietal lobule; ITG = inferior temporal gyrus; IPL = inferior parietal lobule; pFFG = posterior fusiform gyrus; STG = superior temporal gyrus; OFC = orbitofrontal cortex; vIFG1 = ventral inferior frontal gyrus seed 1; THA = thalamus; vIFG2 = ventral inferior frontal gyrus seed 2; PCUN = precuneus; BA = Brodmann area; FD = frame-by-frame displacement: *p*<0.05 corrected.

### RSFC computation and RSFC-behavior correlation analyses

The RSFC analysis was performed using the Resting-State fMRI Data Analysis Toolkit (REST) [25]. We conducted RSFC computation and multiple comparison correction within a grey matter (GM) mask, which was generated using the following procedure. First, voxels in the SPM5 GM mask with a probability higher than 0.4 were included. Second, voxels within the cerebellar regions were discarded using the Automated Anatomical Labeling (AAL) template [26], given the signal distortion in the cerebellum. In total, there were 36, 272 voxels in the GM mask.

For RSFC calculation, the mean time series of each ROI were first computed for each participant by averaging the time series of all the voxels in the ROI. Then the mean time series of each ROI were correlated against every other voxel within the GM mask so as to produce an RSFC *r*-map, in which the *r*-value of each voxel represents the extent to which its activity is synchronized with the ROI. Next, individual *r*-maps were converted into *z*-maps with the application of Fisher's *r*-to-*z* transformation, which yields an approximately normally distributed value that can be used for further statistical analysis.

RSFC-behavior correlation analyses were then conducted to identify those RSFCs whose strength might predict reading performance. Given the high reading accuracy, we used reaction time (RT) as the index of reading competence. Only correct responses were included in RT analyses. We first converted RT into *z*-scores and then obtained their inverse numbers as reading scores so that higher behavioral scores corresponded to more efficient behavioral performance. Subsequently, the RSFC-reading correlation was computed using the “REST Correlation Analysis” command in the REST software, which directly calculated the Pearson's correlation coefficient between the Fisher-*z*-transformed RSFC strength and reading scores for each voxel and produced an *r*-map. These *r*-maps were then corrected for multiple comparisons with AlphaSim (originally in AFNI software and implemented in REST, see the AlphaSim command at http://afni.nih.gov/afni/docpdf/AlphaSim.pdf). The individual voxel threshold was set at *p*<0.01 and a cluster with its volume larger than 783 mm^3^ (29 adjacent voxels) yielded a corrected significance threshold of *p*<0.05.

To rule out the possibility that any significant RSFC-reading correlation relationships may be due to inter-individual differences in the peripheral processes of reading such as visual input detection and output articulation, these RSFC-reading relationships were further examined with participants' performance in the cued articulation task being controlled. The strength of these RSFCs was computed by first extracting the mean time series of a sphere centering on the peak MNI coordinate of voxels that showed significant RSFC-reading correlation relationships with a radius of 6 mm and then correlating this with the mean time series of corresponding ROIs. The RT of the cued articulation task was also transformed into *z*-scores and inversed to obtain the cued articulation scores. Note that recent studies have suggested that the head motion effects need to be scrutinized beyond the application of standard realignment and motion regression analysis strategies [27], [28]. We thus obtained the mean frame-by-frame displacement (FD) and further examined our RSFC-reading effects after regressing out the mean FD values. The mean FD values were defined by the mean absolute displacement of each brain volume compared to the previous volume in translation and rotation in the x (left/right), y (anterior/posterior), and z (superior/inferior) directions. We used mean FD in translation (derived from the following formula) as the measure of head motion given that FD in translation and rotation have been shown to be strongly correlated [28]:
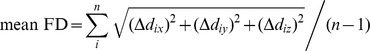
where 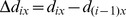, and similarly for the other parameters 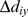 and 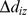, in which *i* = 2….*n*. *i* refers to the i*th* frame of the resting scan and *n* is the total number of frames. We then calculated a partial correlation between the Fisher-*z*-transformed RSFC strength and reading scores when controlling for the cued articulation scores and mean FD. Significance threshold was considered to be *p*<0.05.

### Testing the resting-state network for English reading with Chinese reading data

To more directly examine whether the English-reading-related RSFCs are universal across languages, we carried out a *post hoc* analysis on RSFCs that were found to be significantly correlated with reading competence in adult English readers. Specifically, we tested the extent to which the RSFCs reported in Koyama et al. (2011) for adult English readers could predict reading performance of our Chinese participants. The reading-related RSFCs for adult readers reported in Koyama et al.(2011)'s study came from two types of analyses: a whole-brain analysis based on *F*-test of RSFC-reading relationships in the adult group (four RSFCs were associated with adult reading: L.PreCG-L.SMA, L.PreCG-L.PoCG, L.PreCG-R.PoCG, L.IFGop-L.STG; see Table 3, top half), and further comparisons between adults and children (four additional RSFCs showing adult-child differences and significantly correlating with reading performance in adults: L.FFG-L.IFG, L.FFG-L.IPL, L.FFG-B.PCUN/PCC, L.FFG-B.vMPFC; see Table 3, bottom half). Clearly, the results from the whole-brain analysis in the adult group were more comparable to our results. Nonetheless, to be more conservative and to consider any potential RSFCs relevant to English reading more comprehensively, we also included the RSFCs obtained by the adult-child contrast as ROIs in our analyses. To compute the strength of these RSFCs in our Chinese participants, for each RSFC we first created spheres centering on the reported MNI coordinates of ROI pairs with a radius of 6 mm and then extracted the mean time series for each participant by averaging over voxels within each ROI. Next, the RSFC strength was calculated through direct correlation of mean time series for each ROI pairs. Finally, the Fisher-*z*-transformed RSFC strength was correlated with Chinese reading scores. Significance threshold was considered to be *p*<0.05.

**Table 3:** Correlation relationships between English-reading-related RSFCs and Chinese reading competency.

RSFCs	English reading	Chinese reading
	(Adapted from Koyama et al, 2011)	(Current study)
L.PreCG-L.SMA	*r* = 0.49, *P*<0.05	*r* = 0.002, *P* = 0.99
L.PreCG-L.PoCG	*r* = 0.56, *P*<0.01	*r* = 0.22, *P* = 0.24
L.PreCG-R.PoCG	*r* = 0.65, *P*<0.001	*r* = 0.06, *P* = 0.76
L.IFGop-L.STG	*r* = 0.56, *P*<0.01	*r* = −0.15, *P* = 0.40
L.FFG-L.IFG	*r* = 0.72, *P*<0.001	*r* = 0.02, *P* = 0.91
L.FFG-L.IPL	*r* = 0.65, *P*<0.001	*r* = 0.25, *P* = 0.17
L.FFG-B.PCUN/PCC	*r* = −0.67, *P*<0.001	*r* = 0.05, *P* = 0.80
L.FFG-B.vMPFC	*r* = −0.56, *P*<0.01	*r* = −0.32, *P* = 0.08

Note: L = left; R = right; B = bilateral; PreCG = precentral gyrus; SMA = supplementary motor area; PoCG = postcentral gyrus; IFGop = inferior frontal gyrus opercularis; STG = superior temporal gyrus; FFG = fusiform gyrus; IPL = inferior parietal lobule; PCUN/PCC = precuneus/posterior cingulate cortex; vMPFC = ventral medial prefrontal cortex.

## Results

The mean reaction time of the single-character reading task was 620 ms (SD = 88 ms, range = 473–822 ms). The accuracy of the single-character reading task approached ceiling levels of performance with little variance (mean = 98.60%; SD = 0.87%; range = 96.67–100%).

**Figure 2:**
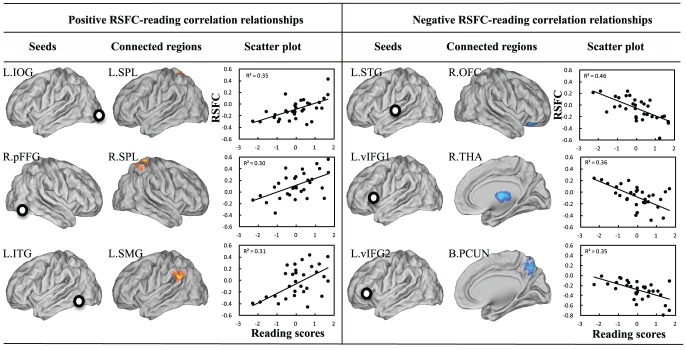
RSFC-reading correlation relationships. RSFC values were computed as the correlation between the mean series of the cluster and the corresponding seed. L = left; B = bilateral; R = right; IOG = inferior occipital gyrus; SPL = superior parietal lobule; ITG = inferior temporal gyrus; IPL = inferior parietal lobule; pFFG = posterior fusiform gyrus; STG = superior temporal gyrus; OFC = orbitofrontal cortex; vIFG1 = ventral inferior frontal gyrus seed 1; THA = thalamus; vIFG2 = ventral inferior frontal gyrus seed 2; PCUN = precuneus: *p*<0.05 corrected.

By correlating the grey-matter RSFC maps of Chinese reading-related ROIs with Chinese reading scores, we found that the strength of the following RSFCs was positively correlated with reading competence (Figure 2 and Table 2): between the L.IOG seed and left superior parietal lobule (L.IOG-L.SPL, cluster-level *r* = 0.59, *p*<0.005), between the R.pFFG seed and right superior parietal lobule (R.pFFG-R.SPL, cluster-level *r* = 0.54, *p*<0.01), and between the L.ITG seed and left inferior parietal lobule (L.ITG-L.IPL, cluster-level *r* = 0.56, *p*<0.05). We also found that the following RSFCs were negatively associated with reading performance: between the L.STG seed and right orbitofrontal cortex (L.STG-R.OFC, cluster-level *r* = −0.68, *p*<0.01), between the L.vIFG1 seed and right thalamus (L.vIFG1-R.THA, cluster-level *r* = −0.60, *p*<0.001), and between the L.vIFG2 seed and bilateral precuneus (L.vIFG2-B.PCUN, cluster-level *r* = −0.59, *p*<0.001).

The mean reaction time of the cued articulation task was 369 ms (SD = 81 ms, range = 268–552 ms). Performance in this task and reading competence showed a weak trend of positive correlation (*r* = 0.24, *p* = 0.19). The average of mean FD across participants was 0.05 mm (SD = 0.02 mm, range = 0.019–0.098 mm). Mean FD and reading competence showed a weak trend of negative correlation (*r* = −0.28, *p* = 0.12), suggesting that poor readers tended to have greater head movements. When controlling for both cued articulation performance and mean FD, the above-observed RSFC-reading relationships remained significant (*p*s<0.003, see Table 2).

Finally, we correlated those previously reported RSFCs for English reading in adults [18] with reading scores of our Chinese participants to test whether these RSFCs were also related to Chinese reading. As shown in Table 3, while the eight RSFCs showed significant correlation with reading competence in adult English readers, none of them were significantly associated with the performance in Chinese single-character reading (*p*s>0.08).

## Discussion

In the current study, we examined the correlation between the RSFC maps of nine Chinese reading-related ROIs and inversed *z*-scores of reaction time in a Chinese single-character reading task and found that reading competence was positively correlated with the RSFC strength between left inferior occipital gyrus and left superior parietal lobule (L.IOG-L.SPL), between right posterior fusiform gyrus and right superior parietal lobule (R.pFFG-R.SPL) and between left inferior temporal gyrus and left inferior parietal lobule (L.ITG-L.IPL), while negatively associated with several RSFCs: between left ventral frontal gyrus and right thalamus and bilateral precuneus and between left superior temporal gyrus and right orbitofrontal cortex. We also found that these RSFC-reading relationships remained significant after the cued-articulation performance was controlled, which suggests that these RSFC-reading relationships cannot be explained by the peripheral processes of the reading task including visual input detection and articulation. Moreover, none of the English-associated RSFCs reported in Koyama et al. (2011) significantly correlated with reaction time of the single character reading task in adult Chinese readers. Below we will discuss about the RSFCs having positive and negative correlation with reading performance, respectively, and then address the language-specific issue in detail.

We found that reading performance was positively correlated with RSFCs between left inferior occipital gyrus (L.IOG) and left superior parietal lobule (L.SPL) and between right posterior fusiform gyrus (R.pFFG) and right SPL. We speculate that the connection between early visual cortices and SPL may contribute to the visuo-orthographic processing of Chinese characters. Unlike most of alphabetic writing systems in which a word is composed of a left-to-right layout of letters and word reading follows a unidirectional scanning path, Mandarin Chinese uses characters occupying two-dimensional space both in height and width as its basic visual input. Therefore, a fine-grained visuospatial analysis is prerequisite to identification of character identity. This notion has been supported by several lines of evidence. Behaviorally, the ability to detect visual spatial relationships was found to predict Chinese character recognition performance in 10 year olds [29]. Many neuroimaging studies on Chinese reading have repeatedly found the activation of SPL [30]–[34], which has been thought to involve in visuospatial analysis [35]–[37]. In addition, a recent study has found developmental increases in bilateral superior parietal lobules when Chinese readers performed spelling and rhyming tasks [38].

Another positive RSFC-reading relationship was observed for the RSFC between left inferior temporal gyrus (L.ITG) seed and left inferior parietal lobule (L.IPL). L.ITG or left fusiform gyrus adjacent to L.ITG has been referred as the visual word form area (VWFA), which specializes in visual word form recognition with striking anatomical reproducibility across individuals [39] and across writing systems [4]. Interestingly, this region has been found to be the highest activated region during Braille reading in blind subjects, suggesting its metamodal nature in reading [40]. One hypothesis regarding the origin of the VWFA holds that the innate connection of VWFA to language-related areas drives this area compared to other parts of the visual ventral stream to develop expertise to abstract word forms [41]. Our finding that the connection between the VWFA and the lateral portion of L.IPL predicted reading competence is consistent with this notion. The involvement of L.IPL in English reading, mainly corresponding to the two largest subregions of IPL (the supramarginal and angular gyri), has been supported by many neuroimaging studies. It has been suggested that supramarginal gyrus is mainly engaged in phonological processing whereas angular gyrus in semantic processing [42]. Most Chinese reading studies have supported its involvement in phonological processing, with some suggesting its role in the correspondence between orthography and phonology [6], and others arguing for its role in short-term maintenance of phonological codes [5], although its role in semantic processing has also been proposed [43]. Taken together, the functional connection between L.ITG and L.IPL in the current study may be associated with coupling between visual-orthographic and phonological processing in Chinese reading, although other functional roles such as semantic access should not be fully excluded.

The RSFC-reading correlation analysis also revealed strong negative correlations between reading performance and RSFCs linking the anterior ventral portion of left inferior frontal gyrus (L.vIFG) and bilateral thalamus (L.vIFG1-B.THAL) and precuneus (L.vIFG2-B.PREC), respectively. L.vIFG has been consistently found to be involved in semantic processing based on both English and Chinese reading studies [6], [44]–[46]. Interestingly, the two spatially adjacent L.vIFG seeds included in the present study showed different RSFC-reading relationships, which might suggest a more fine-grained organization within the L.vIFG region. The thalamus cluster we observed includes the mediodorsal nucleus and the pulvinar and has prominent interconnections with the prefrontal cortex anatomically [47] and functionally [48]. The involvement of the thalamus in the lexical-semantic processing has been suggested via its interaction with prefrontal and temporo-parietal regions [49], [50]. Specifically, the “selective engagement model” [49], [51] proposes that the thalamus serves to selectively modulate the activity and connectivity of cortical areas by attentional allocation so that the more automatic a particular cognitive activity, the less attention it requires, and thus the less vulnerable to thalamic dysfunction. Given the regulatory role of the thalamus in language processing, negative correlation between the L.vIFG-R.THA connection and reading performance may indicate less involvement of language control in adults with higher reading competence when the process is more automatic. As for the precuneus, recent studies have suggested a central role for this region in the default mode network whose activities decrease during cognitive tasks [52], [53]. Thus, we suspect that the negative correlation between the L.vIFG-B.PREC link and reading competence may arise from negative correlation between task-active networks and the default mode network [54].

Another negative RSFC-reading correlation was found in the RSFC between the L.STG seed and right orbitofrontal cortex. The anterolateral region of left superior temporal gyrus has been found to be consistently activated across different writing systems, suggesting its role in phonological identification of word forms [4]. Its connection to left orbitofrontal cortex has been shown both functionally in human beings [55] and anatomically in monkeys [56]. The orbitofrontal cortex has been reported to involve in the encoding of nonverbal abstract auditory information [55]. Thus, the negative RSFC-reading correlation might suggest that reading competence is associated with the inhibition of the auditory stream for nonverbal stimuli so that the brain could be better prepared for automatic phonological processing during reading.

A further note is that the RSFCs showing negative correlation with reading performance discussed here are consistently cross-hemispheric, linking seeds in the left hemisphere and relevant regions in the right. Thus, we speculate that adults with better reading skills might have weaker inter-hemispheric connectivity. This notion is consistent with the right-to-left hemisphere shift in typical reading development as age increases [57]. Dyslexia studies have also found that dyslexic individuals usually showed more right hemisphere activation than normal readers [58], providing further evidence for the associations between reading skills and hemispheric involvement differences.

If we look at the overall patterns of the RSFC-reading correlation studies in English [18] and in Chinese (current study), the convergence is minimal. This, however, does not necessarily mean that different writing systems recruit different neural networks, given that two studies had different focuses with different analysis approaches. While Koyama et al. (2011) focused on developmental trajectories of RSFC-reading relationships by identifying common and distinct connections associated with reading competence in children and adults, we only looked at RSFC-reading correlation in adult readers of Chinese. The discrepancy of results in two studies may be attributed to specific methodological differences or actual cross-linguistic differences. In terms of methodological issues, Koyama et al.'s study measured reading capacity using the accuracy in the Word Reading subtests in Wechsler Individual Achievement Test Second Edition [59] that primarily evaluates phonology-related skills and full-scale Intelligence Quotient (IQ) was controlled as a covariate of no interest. In our study, reading ability was measured using reaction time of the single-character reading task. Moreover, considering that whether IQ should be controlled remains a debatable issue [60], we did not regress out IQ from reading scores. Furthermore, we used “eyes closed” condition for resting-state fMRI data acquisition whereas Koyama et al., (2011) used “eyes open” condition. It has been shown that such differences may affect RSFC patterns of the visual cortex and the default mode network [61], [62]. The result discrepancies in these two studies may also (partly) lie in the differential nature of these two writing systems. Compared to English words, Chinese characters have a simpler syllable structure and have much weaker orthography-phonology correspondence. These differences may explain why in English the effects of the precentral gyrus and left posterior superior temporal region were more salient, as they are likely to be relevant to motor programming in articulation and orthography-phonology conversion, respectively.

Sex differences in language-related skills have been repeatedly reported [63], [64], yet its neural substrate remains elusive. Although the sample size was rather small, we reported the potential gender effects shown by additional ROI analyses separating the participant genders. As can be seen in Table 4, while patterns for most RSFC-reading relationships were similar across the two genders, for two were quite distinct: the association between reading and RSFCs linking left ventral inferior frontal regions and thalamus/precuneus were strong in female readers but was minimal in male readers. Considering the role of L.vIFG in semantic processing, this finding may be related to the neural substrates of gender differences in semantic processing [65] and further investigations with sample size more proper than the current study are warranted.

**Table 4:** RSFC-reading correlation relationships broken down by genders.

RSFCs	*r* (*P*) value of RSFC-reading correlation	
	Male (N = 12)	Female (N = 20)
L.IOG-L.SPL	0.51 (0.09)	0.68 (0.001)
R.pFFG-R.SPL	0.46 (0.13)	0.55 (0.012)
L.ITG-L.IPL	0.44 (0.15)	0.61 (0.004)
L.STG-R.OFC	−0.61 (0.04)	−0.72 (<0.001)
L.vIFG1-R.THA	0.07 (0.83)	−0.74 (<0.001)
L.vIFG2-B.PCUN	0.12 (0.71)	−0.74 (<0.001)

Note: The abbreviations of brain regions are same with Table 2.

One limitation of this study is that the behavioral data were acquired one year after the imaging scanning. Resting-state networks can be modulated through training, even in adults [66], [67] and the exposure to reading may lead to RSFC patterns changes after one year. Nonetheless, Shehzad et al. (2009) have shown that their participants, whose ages (20.5±8.4) were similar to ours (22.4±1.3), showed highly reliable RSFC patterns for both region-of-interest and voxel-wise analyses in both intrasession (<1 h) and intersession (>5 months apart) scanning [68]. Moreover, reading ability has been shown to reach the adult level plateau around 15 years of age [18]. Nonetheless, this issue should be borne in mind during interpretation of our results. In addition, while we found that certain functional connections had significant correlation with Chinese reading, we do not argue that all of them, at least those in the visual cortices (L.IOG-L.SPL, R.pFFG-R.SPL, L.ITG-L.IPL), are specific for reading. It is possible that these functional connections are also relevant for the visual analyses of other types of visual stimuli, such as pictures or faces. Their exact mechanisms warrant further investigation, by comparing their roles in different type of reading tasks, as well as in processing different types of stimuli besides written words. Finally, in the current study we used reaction time of reading single characters as the measure of reading competence in Chinese. It would be useful for future studies to extend our findings to different types of reading competence measures such as accuracy.

## Conclusion

In summary, we mapped out the functional connectivity pattern at rest that related to Chinese reading competence. Specifically, stronger coupling between early visual cortices and bilateral superior parietal lobules and between left inferior temporal gyrus and left inferior parietal lobule was associated with better Chinese reading performance. These connections may be involved in various processes in Chinese character reading, including visuospatial analyses, and phonological/semantic components. Our findings complement the findings on neural correlates of reading on the basis of task-based brain imaging approaches and those with alphabetic languages, and promote further studies to evaluate the precise functional roles of these connections we discovered.
